# CRISPR/Cas Applications in Myotonic Dystrophy: Expanding Opportunities

**DOI:** 10.3390/ijms20153689

**Published:** 2019-07-27

**Authors:** Renée H.L. Raaijmakers, Lise Ripken, C. Rosanne M. Ausems, Derick G. Wansink

**Affiliations:** 1 Department of Cell Biology, Radboud University Medical Center, Radboud Institute for Molecular Life Sciences, 6525 GA Nijmegen, The Netherlands; 2 Department of Human Genetics, Radboud University Medical Center, Donders Institute for Brain Cognition and Behavior, 6525 GA Nijmegen, The Netherlands

**Keywords:** cell therapy, gene editing, gene therapy, muscular dystrophy, myotonic dystrophy, neuromuscular disease, repeat expansion, trinucleotide repeat

## Abstract

CRISPR/Cas technology holds promise for the development of therapies to treat inherited diseases. Myotonic dystrophy type 1 (DM1) is a severe neuromuscular disorder with a variable multisystemic character for which no cure is yet available. Here, we review CRISPR/Cas-mediated approaches that target the unstable (CTG•CAG)n repeat in the *DMPK*/*DM1-AS* gene pair, the autosomal dominant mutation that causes DM1. Expansion of the repeat results in a complex constellation of toxicity at the DNA level, an altered transcriptome and a disturbed proteome. To restore cellular homeostasis and ameliorate DM1 disease symptoms, CRISPR/Cas approaches were directed at the causative mutation in the DNA and the RNA. Specifically, the triplet repeat has been excised from the genome by several laboratories via dual CRISPR/Cas9 cleavage, while one group prevented transcription of the (CTG)n repeat through homology-directed insertion of a polyadenylation signal in *DMPK*. Independently, catalytically deficient Cas9 (dCas9) was recruited to the (CTG)n repeat to block progression of RNA polymerase II and a dCas9-RNase fusion was shown to degrade expanded (CUG)n RNA. We compare these promising developments in DM1 with those in other microsatellite instability diseases. Finally, we look at hurdles that must be taken to make CRISPR/Cas-mediated editing a therapeutic reality in patients.

## 1. Introduction

### 1.1. Basic Principles of CRISPR/Cas Technology

**Figure 1:**
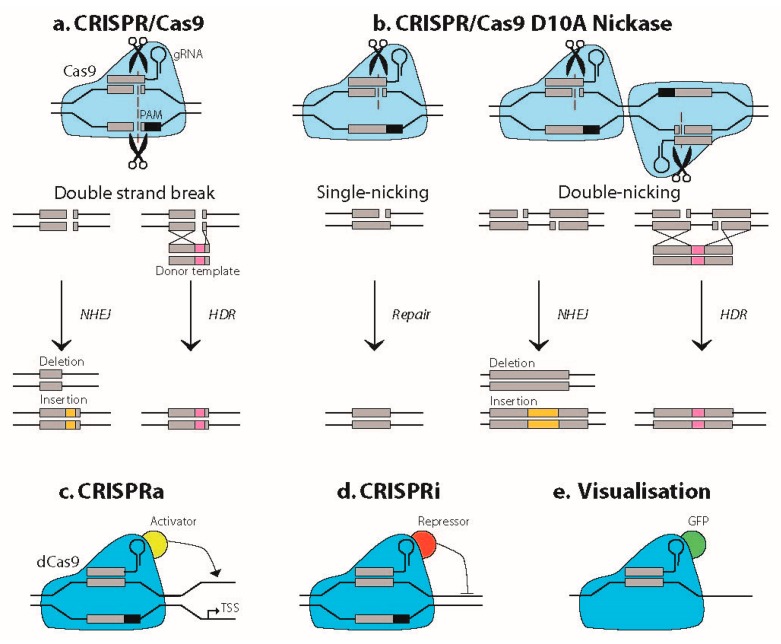
Principles of main CRISPR/Cas9 applications. (**a**) CRISPR/Cas9 generates a double strand break (DSB) in the genome, guided by its small guide RNA (sgRNA) next to a proto-spacer adjacent motif (PAM) sequence. The DSB will in most cases be repaired by error-prone non-homologous end joining (NHEJ), resulting in the formation of an indel. Precise DNA repair will occur through homology-directed repair (HDR) and the use of a suitable donor template in a minority of the cases. (**b**) Cas9 D10A nickase (Cas9n) is mutated in one of its nuclease domains and will therefore introduce a single strand break in the DNA. When two nickases are targeted close to each other, the two nicks effectively generate a DSB, which will be followed by the same repair events as illustrated in a. (**c**–**e**) dCas9 is a double mutant and enzymatically inactive. It is being used as a precise and effective guiding vehicle to the genome and to transcripts. Examples shown here are transcription activation (CRISPRa) and transcription interference (CRISPRi) by dCas9 fusions to transcription activators and repressors, respectively. Fusion of GFP to dCas9 has been used to localize (CUG)n RNA in situ in living cells [6].

Clustered regularly interspaced short palindromic repeats (CRISPR)/CRISPR-associated system (Cas) methodology has a huge impact on research and gene therapy development by its ability to accurately target a specific locus in the genome of eukaryotes [1]. Adapted from an antiviral defense system in prokaryotes, CRISPR/Cas interferes with the genome via a small guide RNA (sgRNA). This sgRNA directs the Cas endonuclease to a DNA target that matches the sgRNA sequence and is located next to the proto-spacer adjacent motif (PAM), a conserved 2–6 base pair DNA sequence bound by Cas itself (Figure 1). Upon binding, the Cas protein will generate a double strand break (DSB) by cleaving the DNA in both strands, which was first shown for Cas9 from *Streptococcus pyogenes* (SpCas9). Although SpCas9 is still widely used, many other Cas proteins have been identified in the meantime, originating from different organisms, each with their own, highly conserved PAM sequence and nuclease characteristics [2,3]. The resultant DSB will commonly be repaired by the cell’s DNA repair system through non-homologous end joining (NHEJ), generally resulting in the formation of an indel at the cleavage site. In a minority of cases, homology-directed repair (HDR) will occur, depending on the cell cycle phase and presence of a suitable donor template. In a research setting or for therapy development, the indel may be used to knockout a protein-coding gene by disturbing its open reading frame, while a HDR strategy may be designed to insert or replace a specific DNA segment (Figure 1).

The application of the CRISPR/Cas system quickly evolved far beyond the initial function of inducing DSBs at desired loci. Mutating one or both of the two nuclease domains of Cas9, respectively, resulted in the generation of Cas9 nickase (Cas9n), which only induces a single strand nick, and catalytically dead Cas9 (dCas9), which bears no nuclease activity at all (Figure 1). dCas9 can block transcription by physically occupying the gene or it may function as a scaffold for fluorophores (e.g., green fluorescent protein (GFP)), transcription activators or inhibitors (i.e., CRISPRa or CRISPRi), and epigenetic modifiers like demethylases and base editors [4,5] (Figure 1). More detailed information on the broad spectrum of CRISPR/Cas technology, beyond the scope of this review, can be found in comprehensive publications on this topic [1,2,3,4,5]. The focus of our review here will be on the use of CRISPR/Cas in the context of research and therapy development for myotonic dystrophy (DM1), a complex, inherited multisystemic disease, caused by an unstable (CTG•CAG)n repeat.

### 1.2. Clinical Genetics and Disease Mechanisms in DM1

DM1 is a severe disorder characterized by diverse symptoms related to a broad range of organs and tissues, including skeletal muscle, heart, brain, eyes and gastrointestinal tract. Symptoms can differ strongly between patients, even within one family. Currently, five subtypes are recognized in the literature, from the mild late-onset, to adult, juvenile and infantile DM1, and finally the severe congenital form of DM (CDM) [7]. The underlying cause of DM1 is a toxic gain-of-function of an expanded (CTG•CAG)n repeat on chromosome 19 [8,9,10]. The repeat length is polymorphic in the human population, generally between 5–23 triplets long. Above a length of 37 triplets, the repeat becomes unstable and can grow to several thousands of triplets. Repeat instability, usually expansion, occurs during life in somatic cells and between generations. Related to this behavior, anticipation is a typical feature in DM1 families, since longer repeats in successive generations correspond to more severe symptoms and earlier age of onset. Exactly how repeat length relates to pathology is still enigmatic. In about 5%–10% of the patients, the (CTG•CAG)n repeat carries imperfections, which is generally associated with a lower expansion rate and a less severe phenotype [11,12].

Expansion of the DM1 (CTG•CAG)n repeat causes toxicity at various levels [8,9,10]. At the DNA level, an expanded repeat forms stable (CTG)n and (CAG)n hairpin-like structures that may cause replication fork stalling during S-phase, leading to cell stress. In addition, repeat-induced chromatin changes, e.g., CpG methylation, may lead to haploinsufficiency of DM1 locus genes, e.g., *DMPK* and *SIX5*. Most experimental evidence, however, points to a toxic function for RNA gene products with an expanded repeat. The DM1 expansion mutation is bidirectionally transcribed and located as a (CTG)n repeat in the 3′ untranslated region (UTR) of *DMPK* and as a (CAG)n repeat in an alternatively spliced intron of the DM1 locus antisense RNA (*DM1-AS*) gene. Expanded *DMPK* transcripts carrying a long (CUG)n repeat are trapped in cell nuclei, where they form abnormal hairpin-like structures that sequester members of the muscle-blind-like (MBNL) family. These (CUG)n RNA-MBNL ribonucleoprotein particles are detected by microscopy as nuclear foci, a hallmark of DM1. Mutant *DMPK* transcripts also stabilize CELF1 (CUGBP-1) by stimulating its phosphorylation. The imbalance in MBNL and CELF1 protein levels, both developmental regulators of RNA processing, leads to aberrant alternative splicing and alternative polyadenylation of many transcripts and abnormal miRNA processing, together resulting in altered expression of a broad range of proteins. Adding to the disturbed proteostasis, the *DM1-AS* (CAG)n repeat is translated by a mechanism called repeat-associated non-AUG (RAN) translation, by which toxic homopolymeric (e.g., polyQ) polypeptides are being formed [13]. In sum, several pathogenic mechanisms likely contribute to disease in DM1. Which of these mechanisms is most dominant, and to what extent tissue- and cell type-specific properties are involved, is not known.

There is no cure for DM1 and treatment is currently limited to disease management [14]. When it comes to therapeutic approaches directed at the cause of disease, several strategies are being tested by different laboratories [15,16,17]. The main three classes of therapeutics are small molecules, antisense oligonucleotides and gene editing technology, including CRISPR/Cas. The CRISPR/Cas system, applied now in a number of publications on the disease, has great potential for DM1, as it may lead to a permanent rescue of cell function. Notably, there’s a second, relatively mild form of myotonic dystrophy, DM2, but to our knowledge, no gene editing studies have been published on this disease. Here, we will present the first steps taken towards gene editing applications in DM1, discuss pros and cons of each approach and reflect on challenges and opportunities. We will compare and learn from recent developments in other microsatellite instability diseases and will define remaining questions. Finally, we will look at hurdles that must be taken to bring CRISPR/Cas-mediated gene editing closer to the patients.

## 2. CRISPR/Cas-Mediated Genome Editing in the DM1 Locus

### 2.1. Excision of the Expanded (CTG•CAG)n Repeat

**Figure 2:**
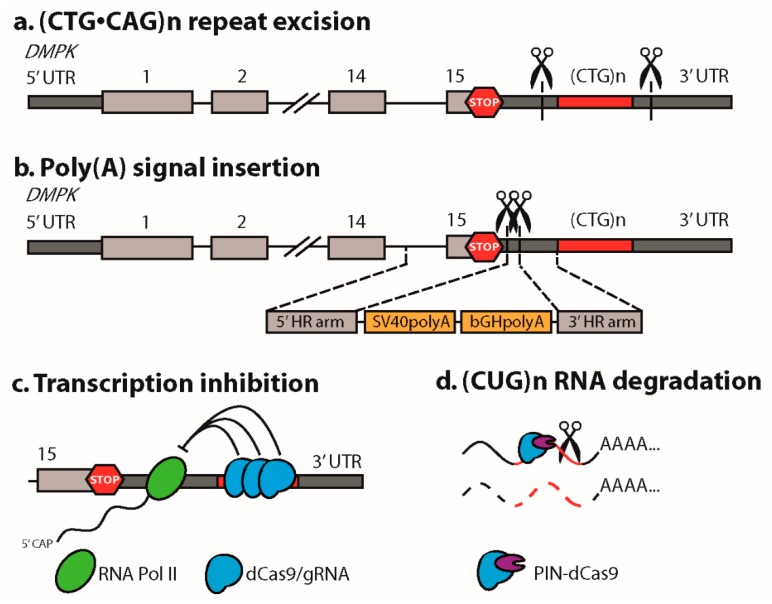
CRISPR/Cas9 strategies successfully applied in DM1 models. (**a**) The (CTG•CAG)n triplet repeat expansion mutation was excised via dual CRISPR/Cas9-mediated cleavage, at either side of the repeat, followed by NHEJ of the two DSBs [18,21,22,23,24]. The *DMPK* gene is shown with its first and last exons, stop codon (stop sign), and the 3′ UTR including the (CTG)n repeat. (**b**) Transcription of the (CTG)n repeat in *DMPK* was prevented by inserting a premature poly(A) signal between the stop codon and the repeat. This was done via a double Cas9n strategy followed by HDR using a donor template [23]. Notably, in this editing strategy the expanded repeat remains present in the genome. (**c**) Recruitment of dCas9 to the expanded (CTG)n repeat in *DMPK* inhibited its transcription by physically blocking RNA polymerase II progression [25]. (**d**) Recruitment of a PIN ribonuclease-dCas9 fusion protein to the (CUG)n repeat resulted in degradation of (CUG)n repeat-containing transcripts [6].

The most straightforward application of CRISPR/Cas9 in DM1, for the first time reported by our own group [18], is to remove the genetic cause of disease by precise excision of the expansion mutation. This was accomplished by designing two sgRNAs targeting flanking sequences at either end of the mutation, followed by joining of the two DSBs through NHEJ, while excluding the repeat-containing fragment in between (Figure 2a). The main advantage of this strategy is that the disease defect is restored at the DNA level, so long repeat-containing transcripts are not produced and downstream toxic effects are eliminated. From a therapeutic point of view, this excision approach is feasible in DM1, because the (CUG)n repeat is not part of the *DMPK* open reading frame, while functional open reading frames in long noncoding RNA *DM1-AS* have not been demonstrated yet [19]. Since expanded (CAG)n repeats in *DM1-AS* transcripts are subject to non-canonical, disease-related RAN translation [20], repeat excision will also abolish the production of toxic homopolymeric proteins.

The success of the repeat excision strategy is confirmed in a number of studies by other laboratories [21,22,23,24]. Together, these reports show the reliability and robustness of the CRISPR/Cas technique, since different choices were made regarding the sgRNA sequences, located closer to or further away from the (CTG•CAG)n sequence (Table 1). To prevent significant perturbation of the 3′ UTR in corrected *DMPK* transcripts, we reasoned that besides the expanded repeat, we should remove as few flanking base pairs from the locus as possible. The design of sgRNAs, however, is being dictated by the presence of a proper PAM sequence (NGG for SpCas9) and the number of predicted off-target effects elsewhere in the genome. In our hands, different sgRNAs, targeting flanking sequences or the repeat itself, demonstrated variable cutting efficiencies and some did not result in any cleavage at all [18]. This may be explained by the complex DNA hairpin structures that can be formed by expanded (CTG•CAG)n repeats [26]. A similar rationale was described by Provenzano et al. (2017), as they speculated that the editing efficiency in regions close to the repeat might be influenced by its abnormal 3D structure [22]. Therefore they chose to target the DM1 locus more distal to the repeat, ~200–300 base pairs up- and downstream. Whether the deleted flanking sequences harbor any regulatory 3′ UTR information for *DMPK* mRNA half-life, translation efficiency or subcellular localization, e.g., through binding of miRNAs or RNA-binding proteins, remains to be investigated.

Regardless of the use of different combinations of two sgRNAs, all reports demonstrate dual cleavage followed by ligation of the two DSBs and exclusion of the repeat segment plus flanking parts. Expanded and unaffected alleles were equally well targeted. Besides, it should be noted that no suitable single nucleotide polymorphisms (SNPs) located near the repeat are available that can be used to discriminate between long and short alleles. In most cases, the new junction precisely matched joining of the two CRISPR/Cas9 cleavage sites. However, small and larger indels at the cleavage sites were also seen, as well as repeat inversions [18,22,23].

Notably, we and others also discovered that a DSB close to the expanded repeat (<50 bp) induces uncontrolled deletion of large repeat segments, thereby resulting in unpredictable repeat contraction [18,21,24]. This phenomenon, not observed when sgRNAs are directed further away from the repeat (>200 bp) [22], relates probably to the occurrence of unstable slipped-strand structures at (CTG•CAG)n tracts in or close to a DSB [27,28] (see for an excellent review on this topic [29]). To us this demonstrates that for reliable and predictable removal of an expanded repeat two highly effective sgRNAs are needed and that single CRISPR/Cas9 cleavage must be avoided. Of note, if one of the two DSBs is repaired by NHEJ whereby an indel is created, this site cannot be cut again and repeat excision is blocked.

Current evidence suggests that the DM1 triplet repeat can be removed from any cell type in the human body, which seems a prerequisite for gene therapy in vivo in a multisystemic disease like DM1. The repeat has been excised in unaffected and DM1 primary and immortalized myoblasts, induced pluripotent stem cells (iPSCs), embryonic stem cells (ESCs), iPSC-derived myogenic cells, iPSC-derived neural stem cells, MYOD1-expressing immortalized fibroblasts, HEK293T cells and transgenic mouse myoblasts (Table 1). Whether CRISPR/Cas9-mediated repeat excision is also possible in terminally differentiated cells like myotubes needs to be investigated. Excision efficiencies may vary between the different cell types and reports, likely depending on the choice of the sgRNAs. We propose, however, that the local chromatin organization surrounding the (CTG•CAG)n repeat does not play a dominant role in cleavage efficiency, given the observation that unaffected as well as expanded, hypermethylated alleles were successfully targeted.

The ultimate goal of removal of the pathogenic repeat in a DM1 patient cell is improvement or preferably reversal of the disease situation. Whether that indeed will be possible in vivo depends on in vivo CRISPR/Cas9 activity, the reversibility of the molecular mechanisms and the resilience of the cells and tissues involved. Well-known biomarkers that are used to measure changes in DM1 disease status at the cellular level are occurrence of repeat RNA/MBNL1 nuclear foci, ratios of certain DM1-typical alternative splice modes, miRNA expression and myogenic differentiation capacity. In most studies, one or more of these molecular measures were tested and these indeed improved after removal of the expansion (Table 1). One particular DNA biomarker for CDM, that deserves special attention, is hypermethylation of the CpG island surrounding the repeat in the DM1 locus [24]. This abnormal chromatin structure is commonly seen in patient cells with repeat lengths of over a few hundred triplets. In a collaborative project between the Eiges laboratory and our group, we compared CpG methylation before and after repeat removal in ESCs and immortalized myoblasts, both carrying CDM-size repeats with corresponding hypermethylation [24]. To our surprise, excision of the repeat in undifferentiated stem cells resets the methylation status in the locus, but methylation levels remain unchanged in affected myoblasts after deletion of the large expansion. These findings suggest a transition from a reversible to an irreversible heterochromatin state by the DM1 mutation, which must be taken into account when considering gene correction in differentiated cells in vitro and in vivo [24].

DNA editing strategies with the purpose to excise a disease-causing repeat have also been designed in other microsatellite expansion disorders. These studies may be informative for therapy development in DM1, although approaches strongly depend on disease-specific features related to (i) the corresponding disease mechanism, i.e., loss- versus gain-of-function; (ii) the location of the unstable repeat in the mutated gene, i.e., in coding or non-coding sequences; (iii) the function of the gene or the repeat sequence itself, i.e., crucial, redundant or insignificant and (iv) the length of the repeat, i.e., many disease-causing microsatellites are relatively short (<100–200 units) compared to the extreme expansions in many DM1 patients.

Like in DM1, the unstable microsatellite in fragile X syndrome (FXS) is a non-coding repeat: A (CGG●CCG)n sequence in the 5′ UTR of *FMR1* on the X-chromosome. A repeat of >200 triplets induces *FMR1* silencing via hypermethylation and chromatin remodeling of the region. Indeed, removal of pathogenic repeats in FXS iPSCs and ESCs was associated with reduced methylation and reactivation of the *FMR1* gene [30,31]. CRISPR/Cas9-mediated editing was accomplished by either a single cleavage 20 bp upstream of the repeat or dual cleavage at either side (~55 bp) of the repeat. Following NHEJ, the entire repeat including short flanking sequences was removed, also in wt alleles (*n* = 9–10).

Another noncoding unstable microsatellite is the (GAA)n repeat in intron 1 of *FXN*, associated with the recessive disorder Friedreich’s ataxia (FRDA), characterized by heterochromatinization of the gene and low protein production. Repeats containing 82 and 190 triplets were excised by dual CRISPR/Cas9 approaches (using *Sp*Cas9 and *Sa*Cas9) and different sgRNA combinations at either side of the repeat (~100–600 bp up and downstream) in transgenic mouse fibroblasts and transfected mouse muscle [32]. (GAA•TTC)n removal raised *FXN* transcript and protein production.

In many (CAG)n expansion diseases, the pathogenic repeat is located in a coding sequence giving rise to the production of proteins with extended polyglutamine (polyQ) stretches. In Huntington’s disease (HD), for example, the pathogenic (CAG)n repeat is located in exon 1 of *HTT*. Dual CRISPR/Cas9 cleavage, ~35 bp up- as well as downstream of the repeat, successfully excised a (CAG)140 repeat plus flanking sequences from a *HTT* transgene in a HD mouse model in vivo [33]. Consequently, excision inactivated the *HTT* transgene, mutant HTT protein was no longer produced and the neurological phenotype in the mice was attenuated. Excision of an expanded (CAG)78 repeat from exon 10 in *ATXN3* in spinocerebellar ataxia type 3 (SCA3) patient-derived iPSCs [34] was also performed through dual CRISPR/Cas9 cleavage (82 bp up- and 11 bp downstream of the repeat). Repeat excision resulted in a premature stop codon in exon 11, but truncated ATXN3 protein was still able to associate with its normal binding partner ubiquitin.

Finally, in an approach to decrease off-target effects and increase specificity, a second report on HD used paired Cas9 nickases, making nicks in the most 5′ CAG triplet and ~60 bp downstream of the repeat, in a series of HD fibroblasts [35]. Both unaffected (CAG)17/21 and expanded (CAG)44–151 repeats were efficiently removed from *HTT*, thereby inactivating these alleles. It remains to be determined whether a paired Cas9n strategy [36,37] will work for typical repeat lengths of hundreds to several thousands of triplets in DM1 [7]. Long distances between pairs of Cas9n may induce slipped strand structures leading to an unpredictable outcome [18,21,24,27,28].

### 2.2. Reduction of the (CTG•CAG)n Repeat Length

An alternative strategy to excision of the expanded repeat followed by error-prone NHEJ, is making use of HDR to precisely repair the expansion mutation and correct the gene (Figure 1). Based on a carefully designed donor template with homology arms, a HDR-mediated strategy can be used to exchange an elongated repeat for a shorter one below the pathogenic threshold, <37 triplets in DM1. In SCA2, Marthaler et al. (2016) reported the correct replacement of 44 for 22 CAG triplets in the open reading frame in exon 1 of *ATXN2* in patient iPSCs [38]. CRISPR/Cas9 and two sgRNAs were used, targeting upstream of the repeat in exon 1 and downstream in intron 1. A comparable correction strategy was successfully performed in HD iPSCs, using two nickases, cutting only ~30 bp apart, ~30 bp upstream of the repeat, to replace 180 for 18 CAG triplets [39]. Unfortunately, a known disadvantage of HDR is its low efficiency and the fact that it is mainly active in proliferating cells [40,41].

An HDR-based strategy to shorten the repeat has not yet been reported for DM1. It is likely that the extreme expansions of hundreds to thousands of triplets represent a complicating factor, since cleavage near the repeat facilitates contractions and rearrangements up to a few kilobase pairs, probably due to the flexibility of repeat hairpin structures [18,21,24]. This type of uncontrollable behavior is supported by earlier data using transcription activator-like effector based nuclease (TALEN) and zinc finger nuclease (ZFN) cleaving short (CTG•CAG)n repeats (<100 triplets) in yeast and human cells, respectively [42,43,44].

A different mechanism is at play when a single strand cut is made inside the repeat with Cas9n [45] (Figure 1). While a DSB may lead to both expansions and contractions, a single strand break is repaired preferably in a repeat-contracting manner. Shorter, stable (CTG•CAG)n repeats in other genes in the genome are likely unaffected by this approach [45]. Reduction of (CTG•CAG)n repeat length by a deliberate single strand cut might be an interesting opportunity to bring an expanded repeat back to a healthy or less harmful size [30], but it is doubtful whether this strategy will be safe enough for in vivo use towards long repeats in DM1.

### 2.3. Allele-Specific Gene Editing

Recent advances in HD paved the way to selectively knock out the mutant, expanded allele, leaving transcription of only the healthy allele [46,47]. By screening the *HTT* locus for SNPs, variants located in PAM sequences for SpCas9 were identified that are specifically linked to the mutant allele. Dual cleavage Cas9 approaches thus excised a ~44 kb fragment, comprising the promoter region, transcription start site and first exons including the (CAG)n repeat [47] or a ~1 kb fragment encompassing part of the promoter and exon 1 including the repeat [46] of mutant *HTT* in patient-derived cells and in a HD mouse model. These allele-selective editing approaches completely abolished or at least reduced mutant *HTT* expression, depending on CRISPR/Cas9 editing efficiency. Future studies could explore the potential of such a personalized approach using SNPs in DM1 too, since complete elimination of expanded *DMPK* transcripts would likely improve DM1 pathology, assuming that DMPK haploinsufficiency is not too harmful in humans [48]. It must be noted, however, that thus far no SNPs specifically linked to the mutant *DMPK* allele have been identified.

### 2.4. Insertion of a Premature Polyadenylation Signal in DMPK

A CRISPR/Cas9-mediated strategy engaging HDR of a non-repeat region in *DMPK* in iPSCs was reported by Xia and coworkers [23]. Rather than focusing on editing the repeat itself, they designed a strategy to prevent transcription of *DMPK*’s (CTG)n repeat. Premature polyadenylation (poly(A)) signals were inserted between the stop codon and the repeat in exon 15 to induce premature termination of RNA synthesis (Figure 2b) [23] (see also [49,50]). A dual Cas9n approach was chosen, introducing two adjacent single strand nicks in opposite strands, to minimize off-target effects. Edited iPSCs and their derivatives lost nuclear foci and splicing abnormalities were repaired. This elegant approach not only avoids the production of toxic (CUG)n RNA, it also releases *DMPK* transcripts with a novel engineered 3′ UTR for translation into the cytoplasm. DMPK haploinsufficiency may thus be alleviated, however the novel 3′ UTR may confer unexpected regulatory features to the engineered *DMPK* transcripts. A significant limitation to this strategy is that the pathogenic repeat remains present in the genome, so detrimental effects on replication stress and heterochromatinization of the locus and silencing of *SIX5* persist. In addition, transcription of the (CAG)n repeat as part of *DM1-AS* will continue, with the risk of the production of RAN translation-mediated homopolymeric proteins [19,20].

## 3. CRISPR/dCas9 as a Guide to the DM1 Locus

By introducing two mutations in the nuclease domains in Cas9, an enzymatically inactive protein was generated, dCas9, that maintained its ability to bind DNA with great precision. The genome guiding ability of dCas9 was applied to neutralize detrimental effects of expanded (CTG•CAG)n repeats without chemically editing the DNA. CRISPR/dCas9 has been shown to interfere with *DMPK* transcription by physically hybridizing to the repeat, thereby blocking passage of RNA polymerase II (Figure 2c) [25]. Of a number of repeat sgRNAs tested, sgRNA (CAG)6, hybridizing to the coding strand, performed best, which is remarkable since CAG is not a favorable PAM sequence for dSpCas9 nor dSaCas9, the two Cas9 enzymes tested. Transcriptional blockade by dCas9 preferentially affected the pathogenic allele, presumably reflecting the occupancy power of multiple dCas9 molecules that can bind the expanded segment. Proof of principle for this strategy was demonstrated in vitro in transfected cells and in DM1 patient cells, while injection of adeno associated viruses (AAVs) expressing dSaCas9 and (CAG)6-sgRNA in *HSA*^LR^ mice, a transgenic DM1 model for DM1 [51], reduced myotonia. Remarkably, no corresponding rescue of *CLCN1* missplicing was observed, presumably due to inefficiency of viral delivery combined with bulk tissue analysis. Evidence was provided that repeat-mediated transcription inhibition also works for other repeat expansion diseases [25].

Ongoing developments in dCas applications have opened up ample opportunities for genome regulation and modulation in DM1 without using endonuclease activity [52]. CRISPRi, for example might be used to target dCas9-repressor fusion proteins to the *DMPK* promoter to impair initiation of transcription, thereby reducing expanded *DMPK* RNA levels (compare the use of CRISPRa in FXS [53]). Unless available SNPs linked to either of the two alleles are used, however, this strategy will not be allele-specific and both *DMPK* genes may be repressed. Other interesting examples are dCas9 fusions to demethylases, to reduce CpG hypermethylation around the expanded repeat and restore normal chromatin structure, or fusion to DNA base editors [54,55], which may introduce interruptions in large (CTG•CAG)n repeats, thereby lowering repeat instability and probably ameliorate the disease phenotype [11,12].

## 4. CRISPR/dCas9-Mediated Targeting and Elimination of Expanded Repeat RNA

CRISPR/dCas9 not only targets double stranded DNA, it is also able to recognize single stranded RNA rather efficiently [49,56]. This principle was used by the Yeo laboratory to visualize expanded (CUG)n transcripts in situ using a dCas9-EGFP fusion protein and a (CAG)n sgRNA in transfected COS cells [6]. Surprisingly, only dCas9 in combination with a (CAG)n guide was able to dissipate nuclear (CUG) RNA foci and reduce repeat RNA expression levels, more or less similar to what has been shown for blocking-type antisense oligonucleotides [57,58]. This effect was strongly enhanced when dCas9 was fused to a PIN-endonuclease domain, providing evidence for an effect at the RNA level, rather than the DNA level as reported by Pinto et al. [25]. A significant drawback of RNA-targeting CRISPR/Cas therapeutics in DM1 is that these must be continuously present, since their activity will not leave a permanent mark in the genome. On the other hand, RNA approaches might have fewer side effects, since complete elimination of off-target transcripts with nearly perfect sgRNA match seems unlikely.

## 5. Therapeutic Outlook for CRISPR/Cas-Mediated Ex Vivo and In Vivo Approaches in DM1

### 5.1. Ex Vivo Cell Therapy

A meaningful therapeutic intervention in DM1 requires gene editing of a large part of the (stem) cell pool to halt disease progression and significantly contribute to improvement in the long term. Therefore, an ex vivo approach, namely cell therapy based on autologous CRISPR/Cas-edited cells from DM1 patients, might be considered.

When focusing on skeletal muscle, the general consensus holds that satellite cells are required for muscle regeneration [10]. However, it is important to realize that also pericytes are indispensable for postnatal growth of skeletal muscle [59,60]. Studies in mice have shown that these cells enter the satellite cell pool, suggesting that they contribute to subsequent regular muscle regeneration [61]. Importantly, it is still not clear whether human pericytes show similar differentiation characteristics and can contribute to skeletal muscle formation in vivo. Since satellite cells have shown problems including poor survival and incompatibility with systemic delivery, pericytes may be considered the preferred choice [10]. CRISPR/Cas-mediated editing efficiency might differ between muscle stem cells (e.g., pericytes, mesoangioblasts, satellite cells) and the further differentiated proliferating muscle progenitors, such as myoblasts.

The use of iPSCs in cell therapy brings two benefits: First, it circumvents the limited proliferative lifespan of primary cells and, secondly, when CRISPR/Cas-mediated editing has taken place, which is never 100% efficient, the correctly edited cells can be selected, clonally expanded and used further [21]. Two laboratories have used DM1 iPSCs both aimed to end up with genetically edited iPSCs [21,23], which can be differentiated into suitable muscle or neuronal progenitor cells (or progenitor cells for other tissues or organs) creating an unlimited amount of autologous, non-immunogenic healthy cells [62]. Repeat-corrected cells will hopefully prove to be useful to halt or at least delay the degenerative process in DM1 patients in the future.

### 5.2. In Vivo Gene Editing

Research on gene editing in the DM1 locus has mainly focused on cell models in vitro in which CRISPR/Cas9 delivery was performed via transfection of expression plasmids and Cas9/sgRNA RNP complexes or via transduction of lentiviruses and AAVs (Table 1) [18,21,22]. In patients, non-viral vector methods seem only applicable for site-specific, local delivery into skeletal muscle [63,64]. Given the multisystemic manifestation of DM1, therapeutic approaches call for systemic delivery via AAV vectors [65,66]. Since it is not possible to select for the correctly edited cells, unlike in cell therapy, the reliability and efficiency of the gene editing process for in vivo gene therapy needs to be exceptionally high. Next to proper selection of sgRNAs and CRISPR/Cas variants, there are important challenges to be considered: (i) Vector size, (ii) vector choice and tissue-specific delivery, (iii) preexisting immune response to the vector and its cargo and (iv) uncontrollable off-target effects [67,68].

The smaller size of AAVs may be beneficial for diffusion into tissues, but requires some creativity with regard to packaging capacity, since only cDNAs up to 5 kb can be accommodated. The coding sequence for SaCas9 is 1 kb smaller than for SpCas9 and fits in an AAV [6] (Table 1). A reported setback might be, however, that the use of SaCas9 induces more repeat inversions [23]. Additional strategies to overcome the size restrictions include the development of hybrid viral capsid structures [69], the use of expression cassettes with small promoters [70], promoter activity derived from the AAV ITR [71] or split AAV vectors, an approach in which a large gene can be split into two parts and separately packaged into two individual split AAV vectors [72].

In recent years, the number of clinical trials in which AAVs have been used for in vivo gene therapy has steadily increased [73]. It still remains challenging to achieve expression of an effective CRISPR/Cas system at therapeutic levels. Especially for DM1, as a multisystemic disorder, many organs must be targeted for a clinical benefit in the patient, e.g., skeletal muscle, heart, gastrointestinal tract and brain. Modification of capsid proteins and incorporation of targeting peptides on the surface of AAV capsids may specify transduction of specific cell types [74]. For example, attempts to improve muscle tropism of AAV2, the best characterized and mostly used vector, led to diverse capsid modifications such as insertion of a seven-amino-acid muscle-targeting peptide [75,76]. Additional target organ specificity could be gained by organ-specific promoters. More specific delivery might be an enormous undertaking, but brings benefits such as decreased vector production and decreased vector exposure.

Furthermore, preexisting immunity is an important issue to consider. First, a recent article identified preexisting immunity against SpCas9 and SaCas9 [77]. Secondly, almost all individuals harbor neutralizing antibodies to AAV due to a prior immune response to naturally occurring viruses [74,78]. The time window, in the first few months of life, during which humans are devoid of any anti-AAV antibodies is narrow and leaves hardly any therapeutic window [79]. Consequentially, AAV-CRISPR/Cas9 can be introduced into patients only once in order to avoid the amplification of the adaptive immune response, thus limiting efficiency to a single dose of the treatment, which might be insufficient [80]. Whilst AAV2 is the best characterized and mostly used vector in clinical studies [76,81], antibodies against the AAV2 serotype are also the most common [82]. To overcome the presence of preexisting immunity against AAV and enable re-administration, researchers are currently using capsid gene shuffling to produce viral variants resistant to neutralizing antibodies [76,81]. Although different serotypes could be used for a second treatment in combination with immune modulatory drugs, selective modification and/or replacement of specific regions of the capsid to create vectors that have lower host immune response seem to be more promising. Selective capsid modifications can be used diversely and will hopefully lead to vectors with more desirable biological properties for infectivity, stability, toxicity and expanded tropism.

As a final point, we need to consider the risk of unwanted off-target cleavage events by CRISPR/Cas9, which is doubled with dual sgRNA use; and structural genomic alterations, like large deletions and inversions. Particularly, unintended germline modifications are a great cause for concern. When treating a multisystem disorder such as DM1, a whole-body activation of CRISPR/Cas9 bears the chance of exposing not only somatic cells to treatment, but also germ cells in the gonads. As discussed by Lander et al. and Monckton, we clearly need to consider how to mitigate and manage risks concerning heritable, non-heritable and unintended heritable genome editing approaches [67,68].

## 6. Conclusions

The exciting power of CRISPR/Cas technology has truly entered the DM1 field and its possibilities for research and therapy development seem virtually endless. Some approaches target and cleave near the unstable repeat mutation in the DNA and are permanent, others are transient and reduce the production or half-life of pathogenic expanded RNA. Several recent additions to the CRISPR/Cas toolbox are waiting to be exploited in DM1 model systems.

Which of these strategies is most effective and safe ex vivo in cells and in vivo in patients cannot be concluded at this moment and more research is warranted. Both the risk of CRISPR/Cas off-target cleavage events—not unique to the DM1 field [83], and unpredictable DNA repair upon cleavage near the unstable repeat—shared with the microsatellite disease community, require serious attention.

Compelling questions that still need to be answered relate to the translation of CRISPR/Cas-mediated strategies to the clinic. Ex vivo and in vivo treatment both have their pros and cons, but which clinical subtype of DM1 should be treated first? What is the best age for treatment; for example in light of the developmental abnormalities occurring in CDM, which likely are not reversible post-natally? Since DM1 is a multisystemic disease, which symptoms, organs or tissue should be considered for therapy or is whole-body treatment really possible and in fact the only beneficial option? Finally, to obtain a clinically meaningful effect, which fraction of cells or part of an organ must be cured? These and many more scientific, clinical, translational and ethical issues should carefully and jointly be discussed by fundamental scientists, clinicians, patients and industry in the coming years to develop the best DM1 therapy possible.

## Figures and Tables

**Table 1:** Study characteristics of CRISPR/Cas approaches in DM1.

Study	CRISPR/Cas Strategy, Cas Type, (CTG•CAG)n Length	sgRNA ^1^	Delivery	DM1 Biomarkers Examined	Cell Types Used
**(CTG•CAG)n Excision**					
Van Agtmaal et al., 2017 [18]	(CTG•CAG)n excision *hSpCas9* Unaffected: *n* = 5/13 DM1: *n* = ~550/2600	Repeat-flanking sgRNAs Upstream: 11 bp Downstream: 51 bp	Nucleofection: Cas9 and sgRNA expression plasmids	*DMPK*, *SIX5* and *DM1-AS* RNA expression DMPK protein expression *DMPK* RNA subcellular distribution (CUG)n RNA foci and MBNL1 foci *BIN-1* ex11 and *DMD* ex78 splicing Myogenic differentiation	Human: Immortalized unaffected and DM1 myoblasts Murine*:* Immortalized DM500 myoblasts
Provenzano et al., 2017 [22]	(CTG•CAG)n excision *eSpCas9* Unaffected: *n* = 5/13 DM1: *n* = 300–1000	Repeat-flanking sgRNAs Upstream: 189 bp Downstream: 305 bp	Lipofection and nucleofection: Cas9 and sgRNA expression plasmids	*DMPK* RNA expression (CUG)n RNA foci and MBNL1 foci *SERCA1* ex22 and *INSR* ex11 splicing Myogenic differentiation DMPK protein expression	Human*:* HEK293FT cells Immortalized unaffected and DM1 inducible *MYOD1*-expressing fibroblasts
Dastidar et al., 2018 [21]	(CTG•CAG)n excision *hSpCas9* Unaffected: *n* = 5/13 DM1: *n* = 1000–1700	Repeat-flanking sgRNAs Upstream: 24 bp Downstream: 51 bp	Lentiviral transduction: CMV-hspCas9-EF1-GFP virus Nucleofection: Cas9/sgRNA RNP complexes	*DMPK* RNA expression (CUG)n RNA foci andMBNL1 foci *SERCA1* ex22 splicing	Human: DM1 iPSC-derived myogenic cells DM1 primary myoblasts DM1 iPSCs
Wang et al., 2018 [23]	(CTG•CAG)n excision *SpCas9* and *SaCas9* Unaffected: *n* = 5 DM1: *n* ≥ 2000	Repeat-flanking sgRNAs Upstream: <220 bp Downstream: <220 bp	Lipofection: Cas9 and sgRNA expression plasmids	(CUG)n and (CAG)n RNA foci	Human: HEK293FT cells DM1 iPSC-derived neural stem cells
Yanovsky-Dagan et al., 2019 [24]	(CTG•CAG)n excision *SpCas9* Unaffected: *n* = 5 DM1: *n* = 2000	Repeat-flanking sgRNAs Upstream: 11 bp Downstream: 47 bp	Transfection: Cas9 and sgRNA expression plasmids	*SIX5* expression DM1 locus CpG hypermethylation H3K9me3 enrichment	Human: HEK293T cells DM1 hESCs
**Insertion Poly(A) Signal Cassette**					
Wang et al., 2018 [23]	Insertion poly(A) signal cassette *SpCas9n* Unaffected: *n* = 5 DM1: *n* ≥ 2000	Paired sgRNAs between stop codon and repeat Donor template: 5′ HR arm: 97 bp 3′ HR arm: 184 bp	Lipofection: Cas9n and sgRNA expression plasmids and donor template	(CUG)n RNA foci *DMPK* RNA subcellular distribution *MAPT* ex3, *MBNL1/2* ex7, *SERCA1* ex22 and *INSR* ex11 splicing Myogenic differentiation	Human: DM1 iPSCs DM1 iPSC-derived neural stem cells
**dCas9-Mediated Repeat Transcription Inhibition, Repeat RNA Visualization and Degradation**					
Pinto et al., 2017 [25]	(CTG)n transcription block *dSpCas9* and *dSaCas9* (CTG•CAG)n plasmids *n* = 0/12/40/240/480/960 (interrupted) DM1: *n* ≥ 2000	(CAG)6 sgRNA	Transfection: plasmids expressing dCas9 and sgRNAs Transduction: AAV2/6-dSaCas9-sgRNA and AAV2/9-dSaCas9-sgRNA	(CUG)n RNA foci (CUG)n RNA expression Multiple splice modes (RNA-seq) RAN translation Expression of (CUG)n and (CAG)n repeat-containing transcripts Myotonia	Human: Transiently transfected HEK293T and HeLa cells DM1 primary myoblasts Murine: *HSA*^LR^ mice (EDL muscle ex vivo, tibialis anterior and gastrocnemius in vivo)
Batra et al., 2017 [6]	(CUG)n RNA visualization and degradation *666dSpCas9*, *dSpCas9-GFP* and *PIN-dSpCas9* (CTG•CAG)n plasmids *n* = 105 *n* = 960 (interrupted) DM1: *n* ≥ 2700	(CAG)n sgRNA	Lipofection: Cas9 and sgRNA expression plasmids Lentiviral transduction: U6-sgRNA and EFS-PIN-dCas9	(CUG)n RNA expression (CUG)n RNA foci MBNL1 foci Multiple splice modes (RNA-seq) Expression of (CUG)n and (CAG)n repeat-containing transcripts	Human: DM1 primary myoblasts Primate*:* COS-M6 cells

^1^ bp upstream/downstream indicate CRISPR/Cas9 cleavage distance in base pairs from first/last triplet in the repeat.

## References

[1] Knott G.J., Doudna J.A. (2018). CRISPR-Cas guides the future of genetic engineering. Science.

[2] Koonin E.V., Makarova K.S., Zhang F. (2017). Diversity, classification and evolution of CRISPR-Cas systems. Curr. Opin. Microbiol..

[3] Komor A.C., Badran A.H., Liu D.R. (2017). CRISPR-Based Technologies for the Manipulation of Eukaryotic Genomes. Cell.

[4] Xu X., Qi L.S. (2019). A CRISPR-dCas Toolbox for Genetic Engineering and Synthetic Biology. J. Mol. Biol..

[5] Sternberg S.H., Doudna J.A. (2015). Expanding the Biologist’s Toolkit with CRISPR-Cas9. Mol. Cell.

[6] Batra R., Nelles D.A., Pirie E., Blue S.M., Marina R.J., Wang H., Chaim I.A., Thomas J.D., Zhang N., Nguyen V. (2017). Elimination of Toxic Microsatellite Repeat Expansion RNA by RNA-Targeting Cas9. Cell.

[7] De Antonio M., Dogan C., Hamroun D., Mati M., Zerrouki S., Eymard B., Katsahian S., Bassez G., French Myotonic Dystrophy Clinical N. (2016). Unravelling the myotonic dystrophy type 1 clinical spectrum: A systematic registry-based study with implications for disease classification. Rev. Neurol..

[8] Sicot G., Gomes-Pereira M. (2013). RNA toxicity in human disease and animal models: From the uncovering of a new mechanism to the development of promising therapies. Biochim. Biophys. Acta.

[9] Thomas J.D., Oliveira R., Sznajder L.J., Swanson M.S. (2018). Myotonic Dystrophy and Developmental Regulation of RNA Processing. Compr. Physiol..

[10] Andre L.M., Ausems C.R.M., Wansink D.G., Wieringa B. (2018). Abnormalities in Skeletal Muscle Myogenesis, Growth, and Regeneration in Myotonic Dystrophy. Front. Neurol..

[11] Cumming S.A., Hamilton M.J., Robb Y., Gregory H., McWilliam C., Cooper A., Adam B., McGhie J., Hamilton G., Herzyk P. (2018). De novo repeat interruptions are associated with reduced somatic instability and mild or absent clinical features in myotonic dystrophy type 1. Eur. J. Hum. Genet..

[12] Braida C., Stefanatos R.K., Adam B., Mahajan N., Smeets H.J., Niel F., Goizet C., Arveiler B., Koenig M., Lagier-Tourenne C. (2010). Variant CCG and GGC repeats within the CTG expansion dramatically modify mutational dynamics and likely contribute toward unusual symptoms in some myotonic dystrophy type 1 patients. Hum. Mol. Genet..

[13] Banez-Coronel M., Ranum L.P.W. (2019). Repeat-associated non-AUG (RAN) translation: Insights from pathology. Lab. Investig..

[14] Ashizawa T., Gagnon C., Groh W.J., Gutmann L., Johnson N.E., Meola G., Moxley R., Pandya S., Rogers M.T., Simpson E. (2018). Consensus-based care recommendations for adults with myotonic dystrophy type 1. Neurol. Clin. Pract..

[15] van der Bent M.L., van Cruchten R.T.P., Wansink D.G., Agrawal S., Gait M.J. (2019). CHAPTER 7 Targeting Toxic Repeats. Advances in Nucleic Acid Therapeutics.

[16] Lopez-Morato M., Brook J.D., Wojciechowska M. (2018). Small Molecules Which Improve Pathogenesis of Myotonic Dystrophy Type 1. Front. Neurol..

[17] Overby S.J., Cerro-Herreros E., Llamusi B., Artero R. (2018). RNA-mediated therapies in myotonic dystrophy. Drug Discov. Today.

[18] van Agtmaal E.L., Andre L.M., Willemse M., Cumming S.A., van Kessel I.D.G., van den Broek W., Gourdon G., Furling D., Mouly V., Monckton D.G. (2017). CRISPR/Cas9-Induced (CTGCAG)n Repeat Instability in the Myotonic Dystrophy Type 1 Locus: Implications for Therapeutic Genome Editing. Mol. Ther..

[19] Gudde A., van Heeringen S.J., de Oude A.I., van Kessel I.D.G., Estabrook J., Wang E.T., Wieringa B., Wansink D.G. (2017). Antisense transcription of the myotonic dystrophy locus yields low-abundant RNAs with and without (CAG)n repeat. RNA Biol..

[20] Zu T., Gibbens B., Doty N.S., Gomes-Pereira M., Huguet A., Stone M.D., Margolis J., Peterson M., Markowski T.W., Ingram M.A. (2011). Non-ATG-initiated translation directed by microsatellite expansions. Proc. Natl. Acad. Sci. USA.

[21] Dastidar S., Ardui S., Singh K., Majumdar D., Nair N., Fu Y., Reyon D., Samara E., Gerli M.F.M., Klein A.F. (2018). Efficient CRISPR/Cas9-mediated editing of trinucleotide repeat expansion in myotonic dystrophy patient-derived iPS and myogenic cells. Nucleic Acids Res..

[22] Provenzano C., Cappella M., Valaperta R., Cardani R., Meola G., Martelli F., Cardinali B., Falcone G. (2017). CRISPR/Cas9-Mediated Deletion of CTG Expansions Recovers Normal Phenotype in Myogenic Cells Derived from Myotonic Dystrophy 1 Patients. Mol. Ther. Nucleic Acids.

[23] Wang Y., Hao L., Wang H., Santostefano K., Thapa A., Cleary J., Li H., Guo X., Terada N., Ashizawa T. (2018). Therapeutic Genome Editing for Myotonic Dystrophy Type 1 Using CRISPR/Cas9. Mol. Ther..

[24] Yanovsky-Dagan S., Bnaya E., Diab M.A., Handal T., Zahdeh F., van den Broek W.J.A.A., Epsztejn-Litman S., Wansink D.G., Eiges R. (2019). Deletion of the CTG Expansion in Myotonic Dystrophy Type 1 Reverses DMPK Aberrant Methylation in Human Embryonic Stem Cells but not Affected Myoblasts. bioRxiv.

[25] Pinto B.S., Saxena T., Oliveira R., Mendez-Gomez H.R., Cleary J.D., Denes L.T., McConnell O., Arboleda J., Xia G., Swanson M.S. (2017). Impeding Transcription of Expanded Microsatellite Repeats by Deactivated Cas9. Mol. Cell.

[26] Petruska J., Arnheim N., Goodman M.F. (1996). Stability of intrastrand hairpin structures formed by the CAG/CTG class of DNA triplet repeats associated with neurological diseases. Nucleic Acids Res..

[27] Axford M.M., Wang Y.H., Nakamori M., Zannis-Hadjopoulos M., Thornton C.A., Pearson C.E. (2013). Detection of slipped-DNAs at the trinucleotide repeats of the myotonic dystrophy type I disease locus in patient tissues. PLoS Genet..

[28] Liu G., Chen X., Bissler J.J., Sinden R.R., Leffak M. (2010). Replication-dependent instability at (CTG) x (CAG) repeat hairpins in human cells. Nat. Chem. Biol.

[29] Mosbach V., Poggi L., Richard G.F. (2019). Trinucleotide repeat instability during double-strand break repair: From mechanisms to gene therapy. Curr. Genet..

[30] Park C.Y., Halevy T., Lee D.R., Sung J.J., Lee J.S., Yanuka O., Benvenisty N., Kim D.W. (2015). Reversion of FMR1 Methylation and Silencing by Editing the Triplet Repeats in Fragile X iPSC-Derived Neurons. Cell Rep..

[31] Xie N., Gong H., Suhl J.A., Chopra P., Wang T., Warren S.T. (2016). Reactivation of FMR1 by CRISPR/Cas9-Mediated Deletion of the Expanded CGG-Repeat of the Fragile X Chromosome. PLoS ONE.

[32] Ouellet D.L., Cherif K., Rousseau J., Tremblay J.P. (2017). Deletion of the GAA repeats from the human frataxin gene using the CRISPR-Cas9 system in YG8R-derived cells and mouse models of Friedreich ataxia. Gene Ther..

[33] Yang S., Chang R., Yang H., Zhao T., Hong Y., Kong H.E., Sun X., Qin Z., Jin P., Li S. (2017). CRISPR/Cas9-mediated gene editing ameliorates neurotoxicity in mouse model of Huntington’s disease. J. Clin. Investig..

[34] Ouyang S., Xie Y., Xiong Z., Yang Y., Xian Y., Ou Z., Song B., Chen Y., Xie Y., Li H. (2018). CRISPR/Cas9-Targeted Deletion of Polyglutamine in Spinocerebellar Ataxia Type 3-Derived Induced Pluripotent Stem Cells. Stem Cells Dev..

[35] Dabrowska M., Juzwa W., Krzyzosiak W.J., Olejniczak M. (2018). Precise Excision of the CAG Tract from the Huntingtin Gene by Cas9 Nickases. Front. Neurosci..

[36] Shen B., Zhang W., Zhang J., Zhou J., Wang J., Chen L., Wang L., Hodgkins A., Iyer V., Huang X. (2014). Efficient genome modification by CRISPR-Cas9 nickase with minimal off-target effects. Nat. Methods.

[37] Ran F.A., Hsu P.D., Lin C.Y., Gootenberg J.S., Konermann S., Trevino A.E., Scott D.A., Inoue A., Matoba S., Zhang Y. (2013). Double nicking by RNA-guided CRISPR Cas9 for enhanced genome editing specificity. Cell.

[38] Marthaler A.G., Schmid B., Tubsuwan A., Poulsen U.B., Engelbrecht A.F., Mau-Holzmann U.A., Hyttel P., Nielsen J.E., Nielsen T.T., Holst B. (2016). Generation of an isogenic, gene-corrected control cell line of the spinocerebellar ataxia type 2 patient-derived iPSC line H271. Stem Cell Res..

[39] Xu X., Tay Y., Sim B., Yoon S.I., Huang Y., Ooi J., Utami K.H., Ziaei A., Ng B., Radulescu C. (2017). Reversal of Phenotypic Abnormalities by CRISPR/Cas9-Mediated Gene Correction in Huntington Disease Patient-Derived Induced Pluripotent Stem Cells. Stem Cell Rep..

[40] Jasin M., Rothstein R. (2013). Repair of strand breaks by homologous recombination. Cold Spring Harb Perspect. Biol..

[41] Heyer W.D., Ehmsen K.T., Liu J. (2010). Regulation of homologous recombination in eukaryotes. Annu. Rev. Genet..

[42] Richard G.F., Viterbo D., Khanna V., Mosbach V., Castelain L., Dujon B. (2014). Highly specific contractions of a single CAG/CTG trinucleotide repeat by TALEN in yeast. PLoS ONE.

[43] Mittelman D., Moye C., Morton J., Sykoudis K., Lin Y., Carroll D., Wilson J.H. (2009). Zinc-finger directed double-strand breaks within CAG repeat tracts promote repeat instability in human cells. Proc. Natl. Acad. Sci. USA.

[44] Mosbach V., Poggi L., Viterbo D., Charpentier M., Richard G.F. (2018). TALEN-Induced Double-Strand Break Repair of CTG Trinucleotide Repeats. Cell Rep..

[45] Cinesi C., Aeschbach L., Yang B., Dion V. (2016). Contracting CAG/CTG repeats using the CRISPR-Cas9 nickase. Nat. Commun..

[46] Monteys A.M., Ebanks S.A., Keiser M.S., Davidson B.L. (2017). CRISPR/Cas9 Editing of the Mutant Huntingtin Allele In Vitro and In Vivo. Mol. Ther..

[47] Shin J.W., Kim K.H., Chao M.J., Atwal R.S., Gillis T., MacDonald M.E., Gusella J.F., Lee J.M. (2016). Permanent inactivation of Huntington’s disease mutation by personalized allele-specific CRISPR/Cas9. Hum. Mol. Genet..

[48] Carrell S.T., Carrell E.M., Auerbach D., Pandey S.K., Bennett C.F., Dirksen R.T., Thornton C.A. (2016). Dmpk gene deletion or antisense knockdown does not compromise cardiac or skeletal muscle function in mice. Hum. Mol. Genet..

[49] Gao Y., Guo X., Santostefano K., Wang Y., Reid T., Zeng D., Terada N., Ashizawa T., Xia G. (2016). Genome Therapy of Myotonic Dystrophy Type 1 iPS Cells for Development of Autologous Stem Cell Therapy. Mol. Ther..

[50] Xia G., Gao Y., Jin S., Subramony S.H., Terada N., Ranum L.P., Swanson M.S., Ashizawa T. (2015). Genome modification leads to phenotype reversal in human myotonic dystrophy type 1 induced pluripotent stem cell-derived neural stem cells. Stem Cells.

[51] Mankodi A., Logigian E., Callahan L., McClain C., White R., Henderson D., Krym M., Thornton C.A. (2000). Myotonic dystrophy in transgenic mice expressing an expanded CUG repeat. Science.

[52] Dominguez A.A., Lim W.A., Qi L.S. (2016). Beyond editing: Repurposing CRISPR-Cas9 for precision genome regulation and interrogation. Nat. Rev. Mol. Cell Biol..

[53] Haenfler J.M., Skariah G., Rodriguez C.M., Monteiro da Rocha A., Parent J.M., Smith G.D., Todd P.K. (2018). Targeted Reactivation of FMR1 Transcription in Fragile X Syndrome Embryonic Stem Cells. Front. Mol. Neurosci..

[54] Eid A., Alshareef S., Mahfouz M.M. (2018). CRISPR base editors: Genome editing without double-stranded breaks. Biochem. J..

[55] Liu X.S., Wu H., Krzisch M., Wu X., Graef J., Muffat J., Hnisz D., Li C.H., Yuan B., Xu C. (2018). Rescue of Fragile X Syndrome Neurons by DNA Methylation Editing of the FMR1 Gene. Cell.

[56] Strutt S.C., Torrez R.M., Kaya E., Negrete O.A., Doudna J.A. (2018). RNA-dependent RNA targeting by CRISPR-Cas9. Elife.

[57] Wheeler T.M., Sobczak K., Lueck J.D., Osborne R.J., Lin X., Dirksen R.T., Thornton C.A. (2009). Reversal of RNA dominance by displacement of protein sequestered on triplet repeat RNA. Science.

[58] Mulders S.A., van den Broek W.J., Wheeler T.M., Croes H.J., van Kuik-Romeijn P., de Kimpe S.J., Furling D., Platenburg G.J., Gourdon G., Thornton C.A. (2009). Triplet-repeat oligonucleotide-mediated reversal of RNA toxicity in myotonic dystrophy. Proc. Natl. Acad. Sci. USA.

[59] Birbrair A., Delbono O. (2015). Pericytes are Essential for Skeletal Muscle Formation. Stem Cell Rev..

[60] Kostallari E., Baba-Amer Y., Alonso-Martin S., Ngoh P., Relaix F., Lafuste P., Gherardi R.K. (2015). Pericytes in the myovascular niche promote post-natal myofiber growth and satellite cell quiescence. Development.

[61] Dellavalle A., Maroli G., Covarello D., Azzoni E., Innocenzi A., Perani L., Antonini S., Sambasivan R., Brunelli S., Tajbakhsh S. (2011). Pericytes resident in postnatal skeletal muscle differentiate into muscle fibres and generate satellite cells. Nat. Commun..

[62] Xia G., Terada N., Ashizawa T. (2018). Human iPSC Models to Study Orphan Diseases: Muscular Dystrophies. Curr. Stem Cell Rep..

[63] Burke C.W., Suk J.S., Kim A.J., Hsiang Y.H., Klibanov A.L., Hanes J., Price R.J. (2012). Markedly enhanced skeletal muscle transfection achieved by the ultrasound-targeted delivery of non-viral gene nanocarriers with microbubbles. J. Control. Release.

[64] Li Y., Wang J., Satterle A., Wu Q., Wang J., Liu F. (2012). Gene transfer to skeletal muscle by site-specific delivery of electroporation and ultrasound. Biochem. Biophys. Res. Commun..

[65] Gregorevic P., Blankinship M.J., Allen J.M., Crawford R.W., Meuse L., Miller D.G., Russell D.W., Chamberlain J.S. (2004). Systemic delivery of genes to striated muscles using adeno-associated viral vectors. Nat. Med..

[66] Arruda V.R., Stedman H.H., Nichols T.C., Haskins M.E., Nicholson M., Herzog R.W., Couto L.B., High K.A. (2005). Regional intravascular delivery of AAV-2-F.IX to skeletal muscle achieves long-term correction of hemophilia B in a large animal model. Blood.

[67] Monckton D.G. (2019). Manage risk of accidental gene editing of germline. Nature.

[68] Lander E.S., Baylis F., Zhang F., Charpentier E., Berg P., Bourgain C., Friedrich B., Joung J.K., Li J., Liu D. (2019). Adopt a moratorium on heritable genome editing. Nature.

[69] Ponnazhagan S., Weigel K.A., Raikwar S.P., Mukherjee P., Yoder M.C., Srivastava A. (1998). Recombinant human parvovirus B19 vectors: Erythroid cell-specific delivery and expression of transduced genes. J. Virol..

[70] Chao H., Mao L., Bruce A.T., Walsh C.E. (2000). Sustained expression of human factor VIII in mice using a parvovirus-based vector. Blood.

[71] Zhang L., Wang D., Fischer H., Fan P.D., Widdicombe J.H., Kan Y.W., Dong J.Y. (1998). Efficient expression of CFTR function with adeno-associated virus vectors that carry shortened CFTR genes. Proc. Natl. Acad. Sci. USA.

[72] Sun L., Li J., Xiao X. (2000). Overcoming adeno-associated virus vector size limitation through viral DNA heterodimerization. Nat. Med..

[73] Galli F., Bragg L., Meggiolaro L., Rossi M., Caffarini M., Naz N., Santoleri S., Cossu G. (2018). Gene and Cell Therapy for Muscular Dystrophies: Are We Getting There?. Hum. Gene Ther..

[74] Nance M.E., Duan D. (2015). Perspective on Adeno-Associated Virus Capsid Modification for Duchenne Muscular Dystrophy Gene Therapy. Hum. Gene Ther..

[75] Yu C.Y., Yuan Z., Cao Z., Wang B., Qiao C., Li J., Xiao X. (2009). A muscle-targeting peptide displayed on AAV2 improves muscle tropism on systemic delivery. Gene Ther..

[76] Wang D., Tai P.W.L., Gao G. (2019). Adeno-associated virus vector as a platform for gene therapy delivery. Nat. Rev. Drug Discov..

[77] Charlesworth C.T., Deshpande P.S., Dever D.P., Camarena J., Lemgart V.T., Cromer M.K., Vakulskas C.A., Collingwood M.A., Zhang L., Bode N.M. (2019). Identification of preexisting adaptive immunity to Cas9 proteins in humans. Nat. Med..

[78] Vandamme C., Adjali O., Mingozzi F. (2017). Unraveling the Complex Story of Immune Responses to AAV Vectors Trial After Trial. Hum. Gene Ther..

[79] Calcedo R., Morizono H., Wang L., McCarter R., He J., Jones D., Batshaw M.L., Wilson J.M. (2011). Adeno-associated virus antibody profiles in newborns, children, and adolescents. Clin. Vaccine Immunol..

[80] Conboy I., Murthy N., Etienne J., Robinson Z. (2018). Making gene editing a therapeutic reality. F1000Res.

[81] Monahan P.E., Samulski R.J. (2000). AAV vectors: Is clinical success on the horizon?. Gene Ther..

[82] Russell D.W., Kay M.A. (1999). Adeno-associated virus vectors and hematology. Blood.

[83] Vakulskas C.A., Behlke M.A. (2019). Evaluation and Reduction of CRISPR Off-Target Cleavage Events. Nucleic Acid Ther..

